# Open Morcellation Through Mini-Laparotomy: A Novel Technique for the Removal of Large Pedunculated Uterine Fibroids Without Full-Length Laparotomy

**DOI:** 10.7759/cureus.88960

**Published:** 2025-07-29

**Authors:** Sampath Gnanarathne

**Affiliations:** 1 Obstetrics and Gynaecology, Faculty of Medicine, University of Peradeniya, Kandy, LKA

**Keywords:** blood loss control, fibroid, mini-laparotomy, morcellation, pedunculated, surgical technique, uterine myomectomy

## Abstract

Removal of large uterine fibroids traditionally requires extended midline laparotomy, leading to significant postoperative morbidity and poor cosmetic outcomes. While laparoscopic surgery offers a favourable outcome, it is not possible in case of large fibroids. Morcellation is a technique used in laparoscopic surgeries, which helps in the disintegration of fibroid into small parts before taking it out.

This technical report describes a technique for removing large pedunculated fibroids via a small infra-umbilical laparotomy using manual morcellation principles. Patients should be selected after mapping of fibroids using MRI and ultrasound scanning. Uterine tourniquet and vasopressin injection before the detachment of fibroid will help to reduce intra-operative bleeding. The mass is progressively peeled inside the abdominal cavity in concentric layers until small enough for extraction through a 4-6 cm incision. This technique allows for safe removal of large fibroids without requiring extended incisions or specialized morcellation equipment, making it ideal for low-resource settings or patients seeking improved cosmetic outcomes.

## Introduction

Uterine fibroids are among the most common benign tumours in women of reproductive age with 25% of women suffering from it [1]. Myomectomy is indicated in most cases due to ongoing symptoms like heavy menstrual bleeding, anaemia and fertility problems [2]. Myomectomies can be performed laparoscopically or as an open procedure. The choice of surgical techniques depends on many factors and the size is one of them. Large fibroids often necessitate surgical intervention when symptomatic or for fertility preservation. The conventional approach to large fibroid removal involves a vertical laparotomy, which is associated with increased pain, blood loss, infection risk, and cosmetic dissatisfaction [3,4]. This is mainly because the surgeon is not able to deliver the large fibroid through a small incision. In laparoscopic surgery, this problem is overcome by morcellating the fibroid into small pieces using a morcellator.

Removal of fibroids, particularly in laparoscopic procedures, can be challenging, especially when dealing with large specimens. Although laparoscopic surgery offers superior postoperative outcomes, the size of the uterus can become a significant limiting factor. A fibroid uterus equivalent to 30 weeks’ gestation poses substantial difficulties in terms of visualization and manipulation, often rendering laparoscopic removal impractical. There are four main methods for specimen extraction during laparoscopic myomectomy: (1) making a small abdominal incision, (2) enlarging the umbilical incision to facilitate removal, (3) placing the myoma in a protective bag within the abdominal cavity and subsequently cutting or crushing it inside the bag, and (4) extracting the myoma through a posterior colpotomy (an incision in the posterior fornix of the vagina) [5,6]. The technique described in this report provides an alternative approach for removing large fibroids that cannot be managed through these standard laparoscopic methods.

This technical note describes a novel technique that mimics laparoscopic morcellation principles through a small infra-umbilical incision to remove large pedunculated fibroids. This approach offers a safe, feasible, and cosmetically favorable alternative to traditional open surgery.

## Technical report

Pre-operative planning

Safe patient selection is essential when planning the described technique, and it should always be the first priority. Comprehensive preoperative assessment with extensive imaging plays an important role in this process. All patients should undergo ultrasound scan (USS) and, where available, magnetic resonance imaging (MRI) for fibroid mapping, which accurately assess fibroid size, location, vascularity, and its relationship to the uterine wall. Imaging will also aid in identifying the presence of multiple fibroids, which is an important limiting factor in employing this technique.

The ideal candidates for this technique are those with large pedunculated fibroid, with no radiological or clinical suspicion of malignancy. These criteria ensure that the procedure can be performed safely and effectively through a limited incision, minimizing patient morbidity while achieving the desired surgical outcome.

Surgical steps

**Figure 1:**
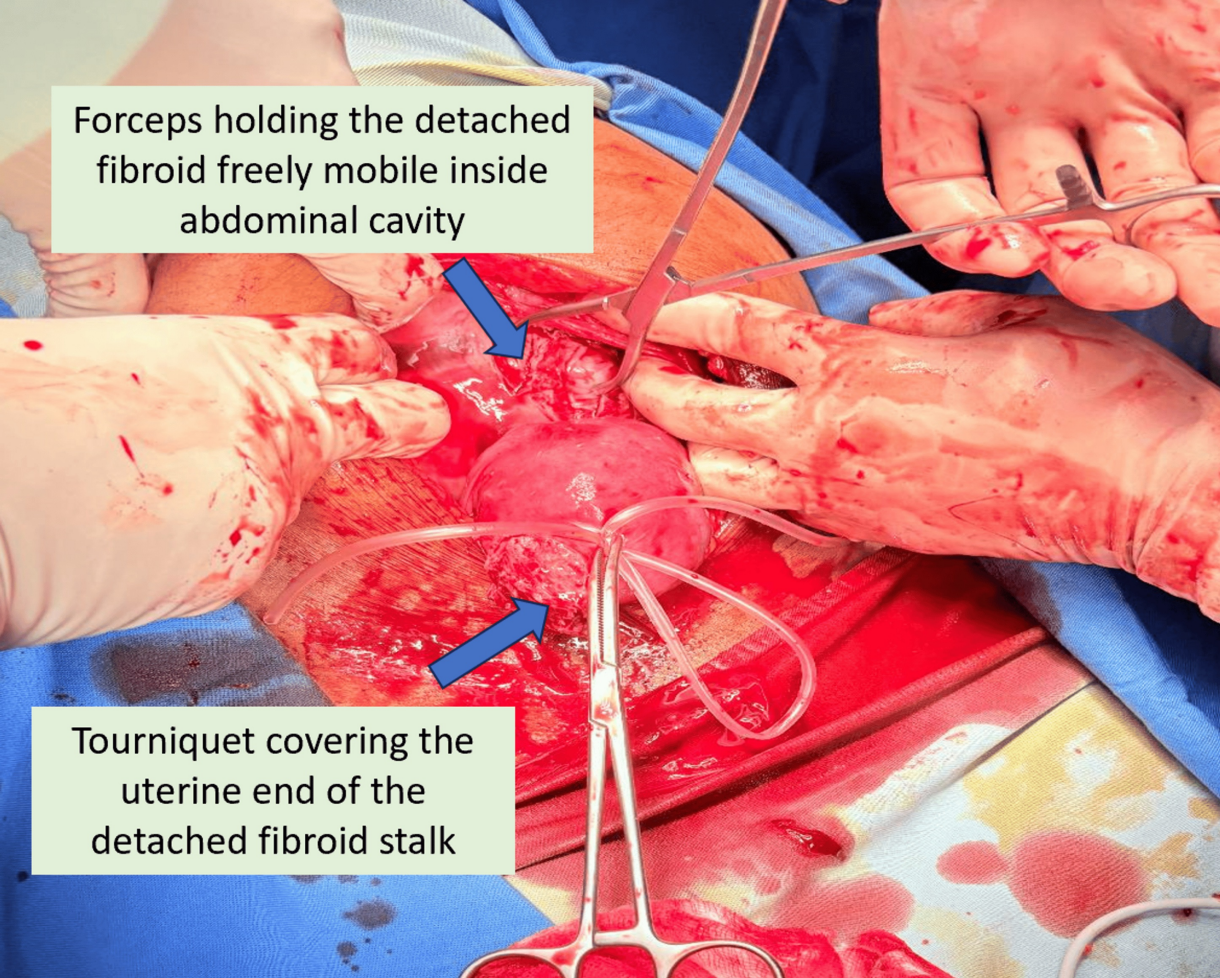
Tourniquet around the fibroid stalk and Mobilization of Detached Pedunculated Fibroid During Mini-Laparotomy The figure illustrates intraoperative appearance  during manual morcellation of a large pedunculated fibroid. A tourniquet is applied at the fibroid stalk base to minimize intraoperative blood loss. The fibroid has been detached from the uterus and is seen held by forceps, freely mobile within the abdominal cavity. This configuration facilitates safe and controlled manual morcellation through a small infra-umbilical incision.

An infra-umbilical mid-line incision measuring approximately 6 cm is first performed to provide access to the abdominal cavity. Once the incision is made, the abdominal wall is retracted either manually by assistants or using self-retaining retractors to achieve adequate exposure. To minimize intraoperative bleeding, hemostatic control is established as early as possible. A tourniquet is placed around the stalk that connects fibroid to the uterine wall. In addition, diluted vasopressin may be injected locally at the base of the fibroid to further reduce blood loss. If vasopressin is contraindicated or not licensed for use, an alternative method involves the administration of 400 mcg of rectal misoprostol approximately three hours prior to surgery to aid in blood loss reduction. The fibroid is then carefully detached by clamping and transecting its pedicle, as shown in Figure 1.

**Figure 2:**
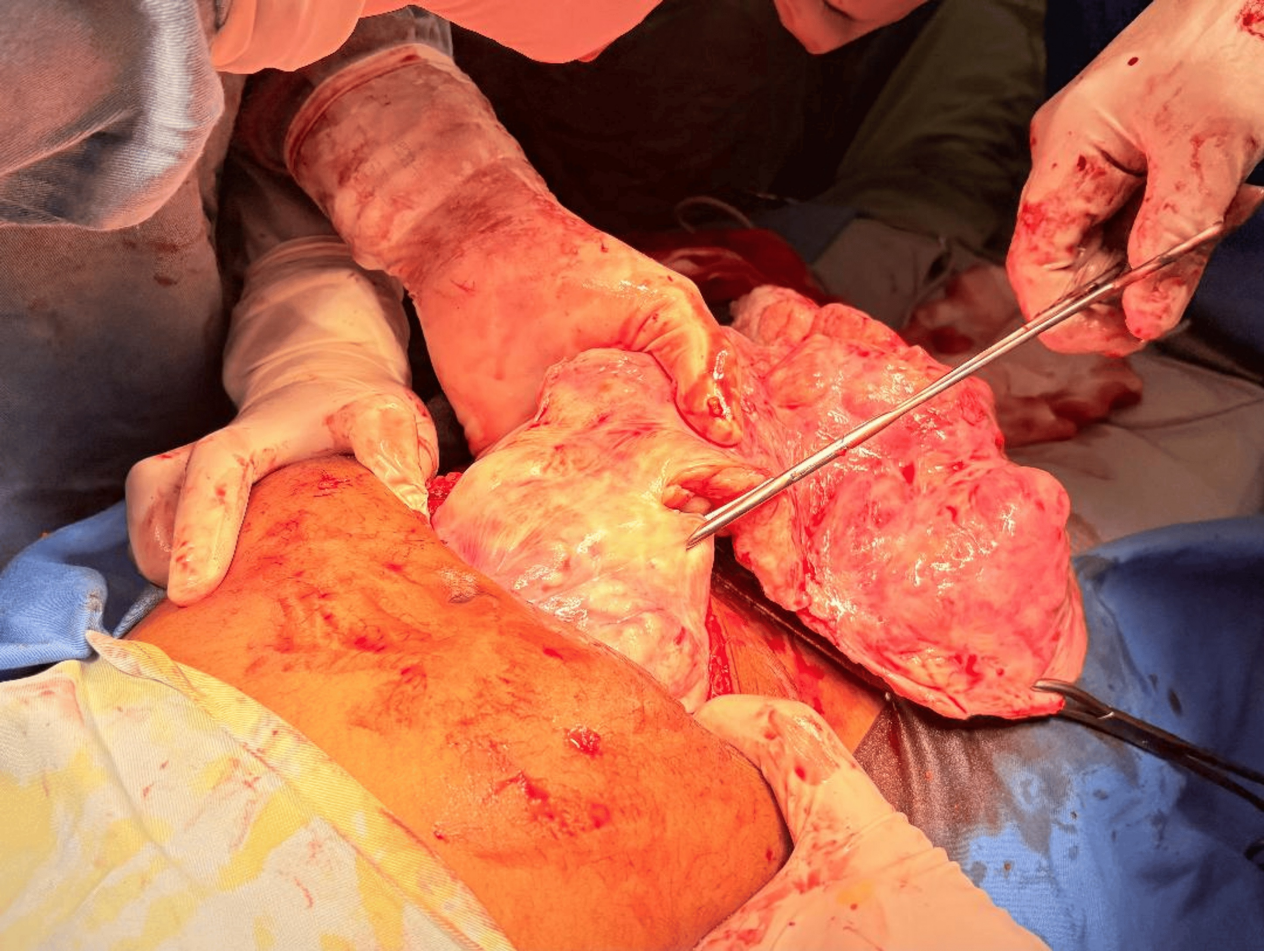
Peeling off the superficial layers of a Freely Mobile Fibroid The figure demonstrates the technique of manual morcellation, where the detached and freely mobile fibroid is progressively peeled in concentric superficial layers using scissors or a scalpel. This controlled dissection reduces the fibroid’s bulk, enabling gradual extraction through a limited infra-umbilical incision. The assistant aids the process by maintaining traction with forceps to allow continuous rotation.

**Figure 3:**
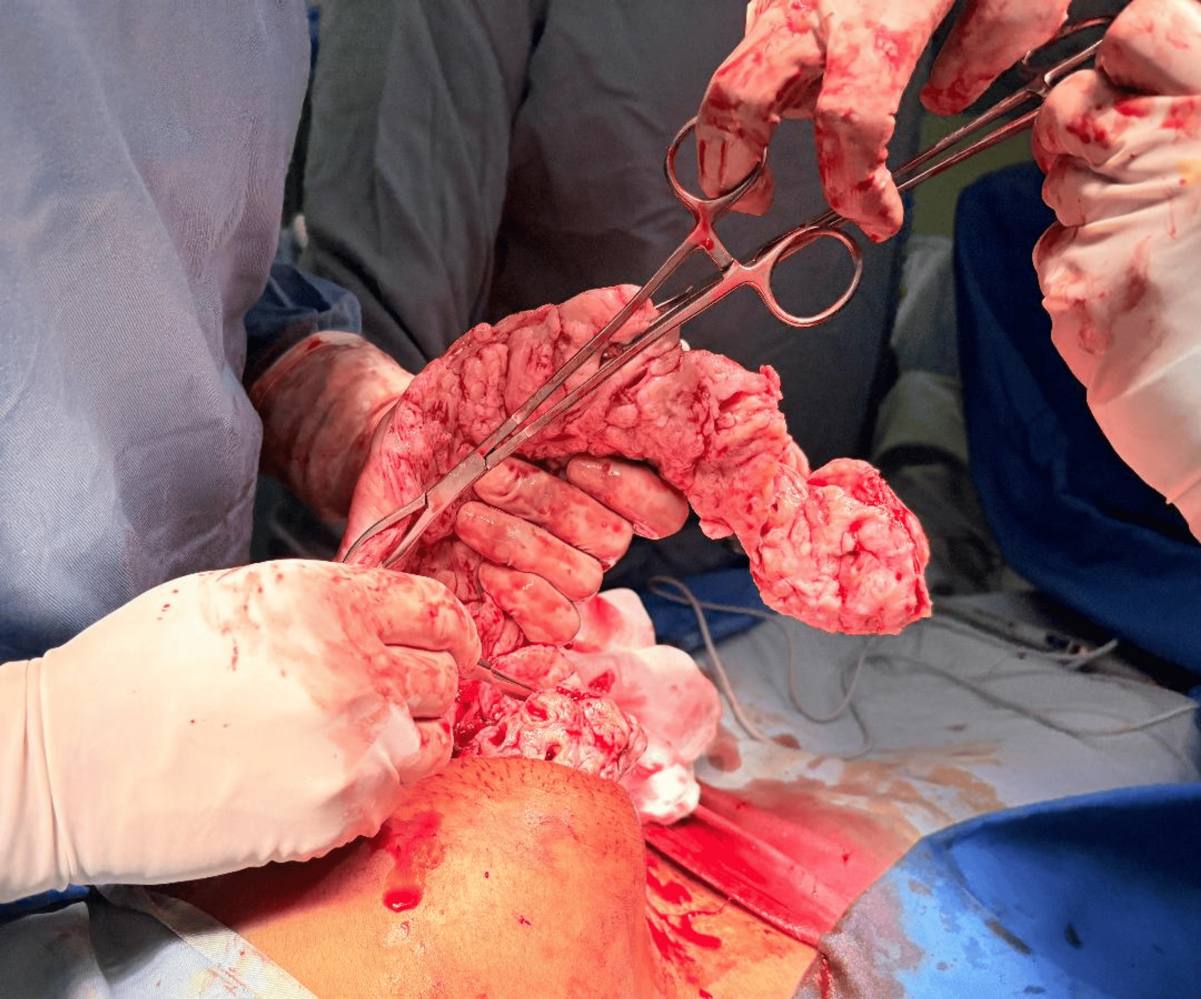
Layered Dissection of Fibroid with Scalpel Under Direct Visualization The image depicts the technique of layered dissection during manual morcellation of a large fibroid. A scalpel is used to carefully shave off thin concentric layers of the fibroid, while the assistant simultaneously rotates the mass using forceps to expose new tissue planes. This method allows controlled size reduction and gradual removal through a small incision. Adequate exposure and clear visibility are essential to maintain precision and ensure safety during the procedure.

Hemostasis at the transection site is achieved by suturing and, when necessary, with the aid of electrocautery. The next step involves manual morcellation of the fibroid. Using a scalpel or scissors, the fibroid is peeled off in layers. During this process, an assistant facilitates concentric peeling by rotating the fibroid using Allis clamps or Moynihan forceps, as demonstrated in Figures 2, 3.

**Figure 4:**
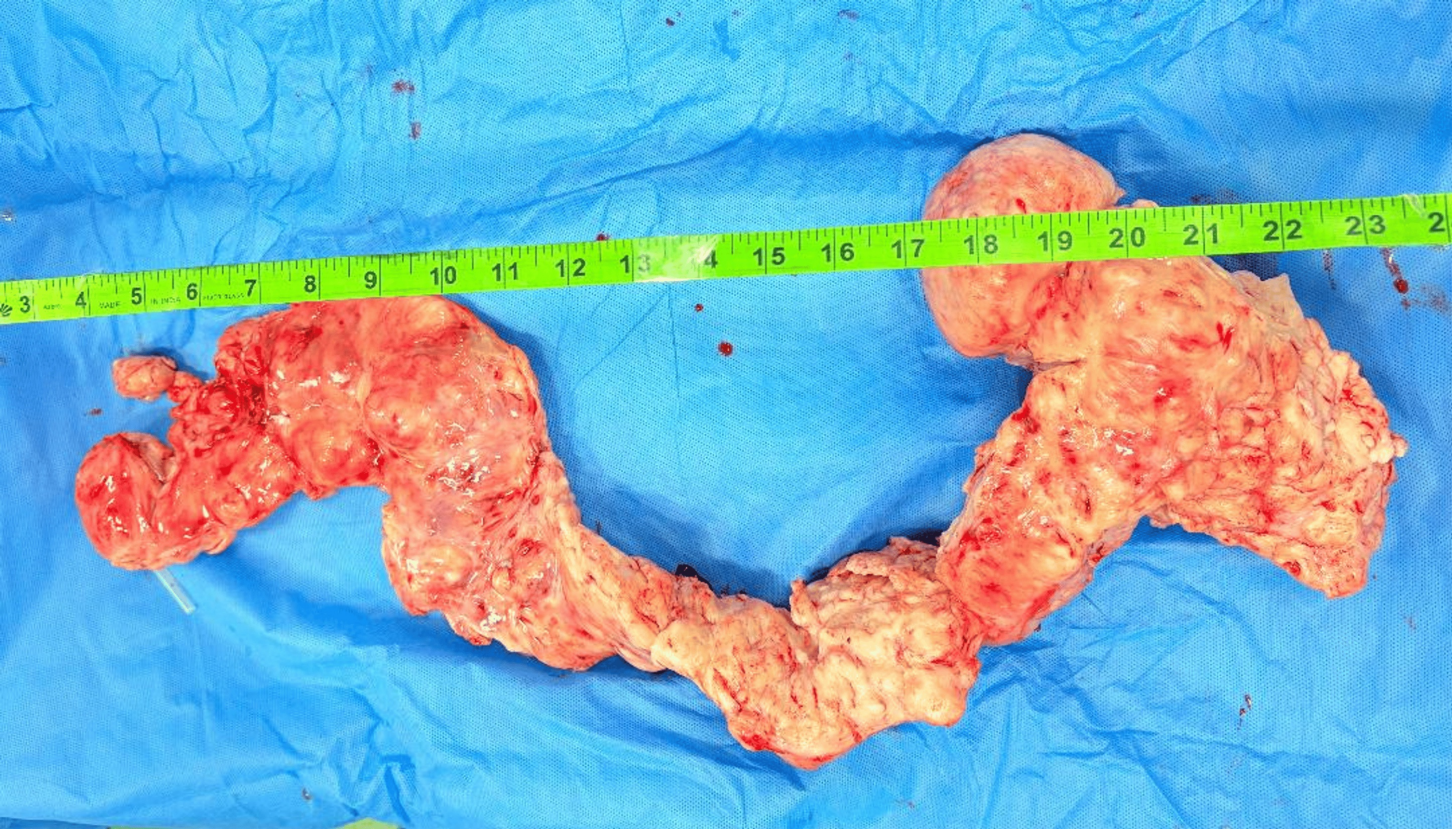
Extracted Fibroid Specimen Following Layered Manual Morcellation The image shows the final appearance of the fibroid specimen after completion of manual morcellation. Instead of retaining its original globular form, the fibroid has been reduced to a long, flattened, sheet-like structure, reflecting the sequential peeling and dissection in layers. This altered morphology demonstrates the effectiveness of the layer-by-layer technique in facilitating the removal of large fibroids through a small infra-umbilical incision.

The dissection continues until the fibroid is sufficiently reduced in size to allow for its complete extraction through the small incision. Any already dissected tissue may be delivered incrementally as space allows. Once the fibroid has been removed, the uterus is elevated toward the incision using gentle traction for reassessment. Any remaining fibroid tissue is excised as needed, and meticulous hemostasis is confirmed. The uterine incision, if not already closed, is sutured at this stage. Figure 4 shows the large fibroid delivered through a small incision.

A video demonstration of the whole procedure is shown in Video 1.

**Video 1:** Step-by-Step Demonstration of the Open Manual Morcellation Technique for Large Pedunculated Fibroids This video provides a step-by-step visual guide to the open manual morcellation technique used for the removal of large pedunculated fibroids through a mini-laparotomy. Key stages include infra-umbilical incision, application of a uterine tourniquet, fibroid detachment, and systematic layer-by-layer dissection using a scalpel under direct vision. The assistant’s role in rotating the fibroid to expose new tissue planes is highlighted, along with techniques to ensure adequate exposure, hemostasis, and safe specimen retrieval. The video aims to serve as a practical resource for surgeons adopting this technique in resource-limited or enhanced recovery settings.

## Discussion

Large pedunculated fibroids, though not easily accessible, can often be enucleated or dissected with relative ease. However, their size frequently necessitates extensive incisions for removal. This technique allows such fibroids to be removed through a small incision by mimicking laparoscopic morcellation, reducing morbidity and improving recovery.

Scalpel morcellation of uterine tissue has been done when there is a difficulty in delivering the specimen in some gynecological surgeries. This is done when the specimen is still inside the Oabdomen or pelvic cavity [7]. In contrast, our techniques involves masses too big to morcellate inside the abdominal cavity. Hence the morcellation is performed outside the abdomen in a systematic manner. The advantage of peeling the fibroid surface act as a safety measure preventing the damage to deep structures like bowel.

In this technique , patient safety is important and therefore patient selection is prioritized. Hemorrhage control is critical; a tourniquet, vasopressin, or misoprostol may be employed to reduce uterine blood flow [8]. Smaller incisions associated with this technique reduces pain and improve cosmetic outcomes. Open morcellation technique eliminates the need for power morcellators, making it suitable for low-resource settings.

One important point to note is that as with other morcellation techniques, thorough preoperative assessment is essential to rule out malignancy. There are evidence that the morcellation techniques are associated with cancer recurrences. Therefore this technique should be avoided [9,10]. This method combines open surgical accessibility with the cosmetic and recovery advantages of minimally invasive techniques.

Various techniques have been advocated for the removal of large fibroids through small incisions in order to minimize surgical morbidity. One study describes the use of a transverse incision combined with a self-retaining atraumatic wound retractor, followed by morcellation of the fibroid, a method the authors reported as successful. The dissection of the fibroid has been done using laparoscopic technique [11]. In contrast, our technique utilizes a open technique with a vertical incision, with dissection performed outside the uterus under direct vision. This approach enhances safety by reducing the risk of injury to internal organs. However, the method used in this study can also be applied to medium-sized fibroids where laparoscopic dissection and enucleation are feasible. In such cases, the fibroid can be exteriorized through a small incision and subjected to open morcellation outside the abdominal cavity. This approach combines the advantages of minimally invasive surgery with the practicality of controlled tissue retrieval, while avoiding the risks associated with intraperitoneal morcellation.

Some authors have described similar size-reduction methods to facilitate vaginal retrieval of fibroids that are relatively small but still too large to be removed through a 2-3 cm posterior fornix incision. In such cases, the "apple-peel" technique has been used to debulk the fibroid [12]. This method involves a comparable superficial peeling approach; however, to our knowledge, no other detailed descriptions of this specific technique for large fibroid removal have been published.

## Conclusions

Manual open morcellation through a mini-laparotomy offers a safe, reproducible, and practical solution for the removal of large pedunculated fibroids without the need for full-length abdominal incisions or specialized morcellation devices. Conventionally, large fibroids were removed through long midline incisions. These incisions extending from xiphisternum to symphysis pubis are associated with increased postoperative pain, delayed wound healing, higher risk of infection, delayed mobilisation, prolonged hospital stays, and unsatisfactory cosmetic outcomes. Such factors may negatively affect the overall recovery experience of the patient. The technique described in this report addresses these concerns by enabling the gradual reduction of fibroid volume through concentric layer-by-layer dissection using basic surgical instruments. This approach allows successful delivery of large fibroids through a small infra-umbilical incision, significantly minimizing tissue trauma. The technique is particularly beneficial in resource-limited settings where access to powered morcellators is restricted, or in patients who prioritize enhanced recovery and minimal scarring.

Appropriate patient selection plays a central role in the success of this method. Pedunculated fibroids, which are attached to the uterus by a narrow stalk, are ideal candidates as they can be detached easily without disturbing the integrity of the uterus. Preoperative imaging and mapping should confirm the size, extent, and the extent of the connection to the uterus. Extra caution should be there to exclude any features suggestive of malignancy, as open morcellation is not suitable in cases where uterine sarcoma or other malignancies are suspected due to the risk of dissemination and recurrence. When applied in suitable cases, this technique may offer comparable outcomes to laparoscopic morcellation while avoiding its equipment requirements and regulatory limitations. It also holds the potential to improve patient satisfaction, facilitate earlier ambulation, and reduce healthcare costs. In conclusion, consideration of adopting this technique by other surgeons based on the detailed steps outlined in this technical note will optimize the management of large fibroids in selected patients and will also aid in refining the technique.
